# Who pays more for early starts? Early-morning time pressure, sleep-related states, and role-contingent work engagement among Chinese university teachers

**DOI:** 10.3389/fpsyg.2026.1824905

**Published:** 2026-08-05

**Authors:** Shuai Teng, Mengqi Sun, Yueying Zhu, Tingting Wang

**Affiliations:** 1 School of Education, Weifang University, Weifang, China; 2 Department of Information and Control Engineering, Yantai Automobile Engineering Professional College, Yantai, China; 3 Department of Students Affairs, Shandong University of Science and Technology, Qingdao, China; 4 College of Computer Science and Artificial Intelligence, Ludong University, Yantai, China

**Keywords:** early-morning time pressure, morning fatigue, role demands, sleep quality, university teachers, work engagement

## Abstract

**Background:**

Early-morning work schedules may act as temporal job demands by reducing recovery opportunities. This study examined associations among early-morning time pressure, sleep-related states, and work engagement in Chinese university teachers.

**Methods:**

A cross-sectional, multi-regional online survey was conducted among 516 university teachers from 30 provincial-level regions in China. Early-morning time pressure was indexed by weekly early-duty frequency, institutional advance-arrival requirements, and actual advance-arrival behavior. Participants also reported prior-night sleep quality, morning fatigue, and UWES-9 work engagement. Analyses were conducted using SPSS 22.0, principal component analysis, and PROCESS Models 6, 7, and 14.

**Results:**

The three indicators showed sufficient convergence to support a composite early-morning time pressure index. Greater time pressure was directly associated with lower work engagement but also showed positive indirect associations through better-rated sleep quality, lower fatigue, and their serial pathway, indicating inconsistent mediation. The time pressure–fatigue association varied by professional rank and administrative responsibilities; age-related moderation was weaker, and teaching experience was not significant. Administrative responsibilities also strengthened the negative association between fatigue and engagement.

**Conclusion:**

Although the cross-sectional self-report design precludes causal inference, the findings suggest that universities should assess early schedules by their total time cost, distribute advance-arrival demands more equitably, and avoid combining early teaching with intensive administrative duties.

## Introduction

### Early-morning scheduling as a temporal job demand

Early-morning work schedules can function as temporal job demands by compressing recovery opportunities and shifting effort investment into the pre-work period. In higher education, 8:00 a.m. classes are a common timetabling practice, but their teacher-side time costs remain underexamined (Yeo et al., 2023a). Although administratively efficient, early starts may constrain sleep opportunity and shape next-day alertness, mood, and performance (Valladares et al., 2023). Research on work timing and recovery suggests that frequent early starts can compress recovery opportunities, and that this burden may be especially consequential when early schedules are accompanied by additional pre-work or pre-class requirements (Weston et al., 2024; Zerbini and Merrow, 2017).

Evidence on early start times has developed most clearly on the student side. Recent work has shown that early-morning classes among university students are associated with sleep-related outcomes and academic functioning, supporting a schedule-alignment lens for understanding how timetables translate into educational consequences (Yeo et al., 2023b). However, this line of research has paid comparatively less attention to university teachers, even though growing evidence suggests that their recovery, well-being, and day-to-day work states are closely tied to burnout risk, work engagement, and the sustainability of academic work (Pakdee et al., 2025; Soutukorva et al., 2025; Wang and Liu, 2023).

### Unequal allocation and measurement of early-morning burden

Focusing on teachers is important for two reasons. First, early-morning teaching involves more than an earlier instructional slot. Teaching work typically includes lesson preparation, course organization, and other non-instructional tasks that can compress recovery windows and reorganize daily routines, especially when these demands accumulate around the start of the workday (Creagh et al., 2025; Mushabe et al., 2022; Roenneberg et al., 2012). Second, early-morning exposure and related preparation demands may not be evenly distributed across staff. Contract status and academic category can shape bargaining power and access to preferred timetables; teaching-focused or junior faculty may carry heavier scheduled teaching loads and have less control over class allocation; administrative appointments can add coordination, meeting, and accountability demands to the same morning window; and disciplinary, laboratory, or practice-based teaching requirements may further restrict scheduling flexibility (Lebovitz et al., 2023; Mudrak et al., 2018; O’Meara et al., 2019). Consequently, an identical 8 a.m. teaching slot may impose different effective time costs across faculty groups, creating institutional time inequality within the same university (Lebovitz et al., 2023; Lee et al., 2025; O'Meara et al., 2017).

A further limitation of teacher-focused discussions is that early-morning burden is often operationalized using a single indicator, such as whether one has an early class or how often early classes occur. Frequency is informative but may underestimate lived intensity (Ballet and Kelchtermans, 2009; Kyndt et al., 2014; Wang et al., 2025). In practice, early-morning burden reflects at least three intertwined elements: (a) exposure frequency, (b) institutional constraints, and (c) teachers’ actual time investment. To better capture this ecological structure, we conceptualize early-morning time pressure as a composite construct integrating weekly early-morning duty frequency, institutional advance-arrival requirements, and teachers’ actual advance-arrival behavior. We also evaluate whether these indicators empirically converge to justify their aggregation into a coherent time–pressure dimension (Bakker and Demerouti, 2007).

### Sleep-related pathways, engagement, and role contingencies

From a recovery and occupational readiness perspective, early-morning time pressure is likely to operate through proximal sleep- and energy-related correlates (Bakker and de Vries, 2021). Subjective sleep quality reflects perceived restoration on the night preceding early teaching and can shape next-day motivational readiness (Kühnel et al., 2017). Morning fatigue captures depletion-related feelings upon arriving at work and may constrain the attentional and energetic resources needed for sustained instructional involvement. These proximal states are therefore plausible correlates linking early-morning time pressure to teachers’ occupational functioning (Madigan and Kim, 2021).

From a work and organizational psychology perspective, early-morning teaching can be understood within the Job Demands–Resources (JD-R) model as a temporal job demand that constrains recovery opportunity and shifts effort investment into the pre-work period (Demerouti, 2025). Effort–recovery and related perspectives further suggest that when work timing compresses nonwork time, individuals may enter the workday with reduced energetic resources, increasing vulnerability to fatigue and undermining motivational states such as engagement (Demerouti et al., 2001; Sonnentag and Fritz, 2007; Zhang and Deng, 2025). In this sense, early starts are not merely “earlier work”; they represent a structural reallocation of time that can alter the balance between recovery processes and work demands.

Importantly, schedule demands rarely operate in isolation. In role-structured work settings, time pressure is often layered with additional responsibilities, accountability demands, and coordination work, thereby creating compounded exposure (Kahn et al., 1964; Tamir and Machovcová, 2025). In universities, administrative responsibilities may bundle managerial tasks with teaching demands, particularly in the early morning, when time is limited and coordination needs are high (Shen et al., 2025). This role–schedule coupling perspective implies that fatigue may be more consequential for engagement when teachers must simultaneously meet instructional and managerial expectations, motivating a role-contingent test of the fatigue–engagement link (Floyd, 2016; Chaaban et al., 2025).

Work engagement is a central outcome for evaluating the sustainability of teaching practices under time-compressed schedules. As a motivational state characterized by vigor, dedication, and absorption, engagement is sensitive to recovery conditions and daily energy availability and is closely related to instructional vitality and longer-term occupational well-being (Bakker and de Vries, 2021). Accordingly, early-morning time pressure may be associated with teachers’ work engagement both directly and indirectly through sleep-related appraisals and morning fatigue (Granziera et al., 2021).

### The present study

To integrate these relationships within a single explanatory framework, the present study draws primarily on the JD-R model. In the proposed framework, early-morning time pressure is treated as a temporal job demand, subjective sleep quality and morning fatigue are treated as proximal functioning states, and work engagement is treated as the primary occupational outcome. The JD-R framework also suggests that the effects of demands are unlikely to be uniform across workers. Role structure and career positioning may alter how temporal demands are experienced and translated into strain (Shen et al., 2025; Wang et al., 2025). In the present study, age, teaching experience, professional rank, and administrative responsibilities are examined as potential boundary conditions, with particular attention to whether administrative responsibilities intensify the extent to which morning fatigue is associated with lower engagement.

Importantly, the early-morning pressure–recovery association may not be uniformly negative. Teachers facing predictable early-start demands may adopt anticipatory adjustments, such as stricter evening routines, to ensure readiness. Such adjustments could preserve perceived sleep quality despite elevated time pressure (Lee, 2024; Völker et al., 2026). At the same time, these adjustments may coexist with broader reductions in time autonomy or recovery opportunities, making it important to examine both indirect and direct associations in the same framework.

**Figure 1:**
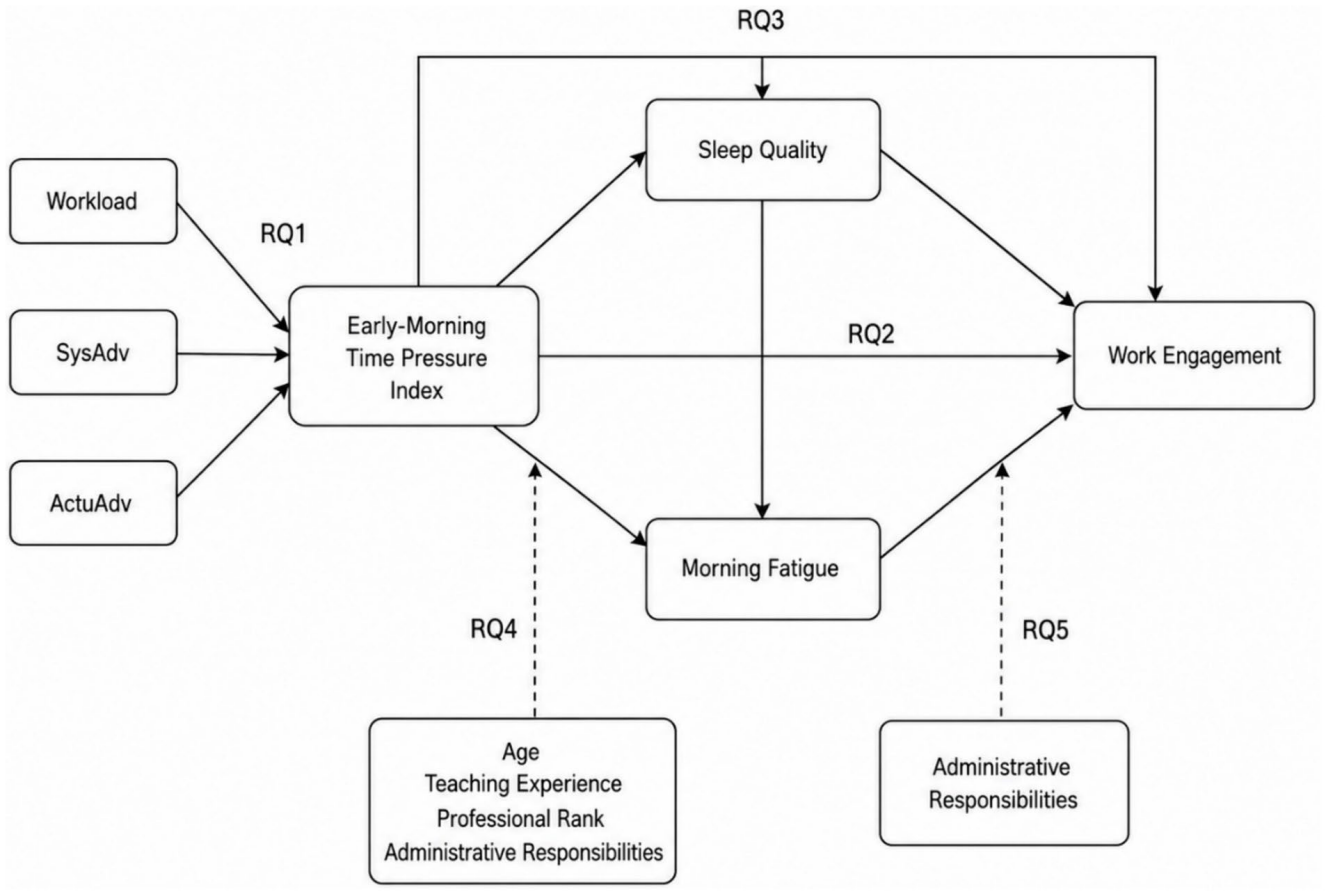
Proposed conceptual framework. Workload, SysAdv, and ActuAdv represent weekly early-duty frequency, institutional advance-arrival requirements, and actual advance-arrival behavior, respectively. Solid arrows indicate the focal direct and indirect associations, whereas dashed arrows indicate moderation effects. RQ1 concerns the convergence of the three indicators into the Early-Morning Time Pressure Index; RQ2 concerns the association between the Index and work engagement; RQ3 concerns the indirect pathways through sleep quality and morning fatigue, including the serial pathway; RQ4 concerns moderation of the Index–morning fatigue association by age, teaching experience, professional rank, and administrative responsibilities; and RQ5 concerns moderation of the morning fatigue–work engagement association by administrative responsibilities. The arrows represent the proposed analytical relationships and do not imply causality.

Motivated by concerns about schedule-related job demands and role-based time–cost allocation in higher education, the present study addresses five research questions. RQ1 asks whether weekly early-duty frequency, institutional advance-arrival requirements, and teachers’ actual advance-arrival behavior empirically converge to support a composite early-morning time pressure index. RQ2 asks whether early-morning time pressure is associated with work engagement among university teachers. RQ3 asks whether subjective sleep quality and morning fatigue are statistically implicated in this association, including a serial pathway from time pressure to sleep quality to fatigue and then to engagement. RQ4 asks whether the association between early-morning time pressure and fatigue varies as a function of age, teaching experience, professional rank, and administrative responsibilities. Finally, RQ5 asks whether administrative responsibilities further condition the association between fatigue and work engagement. By framing early starts as a temporal job-demand issue rather than a simple scheduling inconvenience, the study seeks to contribute to theory on work timing and recovery-related functioning and to evidence relevant for role-sensitive work design and scheduling practice in higher education. To clarify the proposed relationships among the study variables, the conceptual framework is presented in Figure 1.

## Method

### Study design and procedure

This cross-sectional study used an online survey in China to examine associations among early-morning time pressure, sleep-related states, morning fatigue, and work engagement among Chinese university teachers. Data were collected in November 2025 via Wenjuanxing (Questionnaire Star). The survey link was distributed through WeChat and faculty-related networks. The first page provided study information and an informed-consent statement; only consenting participants proceeded. Responses were anonymous and no personally identifiable information was collected.

The questionnaire first assessed demographic characteristics and general early-morning teaching arrangements. It then asked respondents to recall the most typical early-morning (“early-start”) teaching situation they had experienced during the previous week and to answer the subsequent sleep-, fatigue-, and work-related items with reference to that focal episode. This instruction was used to anchor the state-oriented measures in a concrete and temporally proximal early-morning work context rather than in general impressions. The corresponding instruction in the questionnaire explicitly stated: “Please recall the most typical ‘early-eight’ situation during the past week and answer the following questions based on that day’s experience.”

### Participants and sampling

Convenience sampling was used to recruit in-service university teachers across China. A total of 526 responses were received; after excluding cases without consent, with substantial missing data, or with logically inconsistent responses, 516 questionnaires were retained (effective rate = 98.1%). Participants represented 30 provincial-level regions. Given the non-probability sampling design, representativeness cannot be assumed.

## Measures

The questionnaire included demographic characteristics, early-morning teaching arrangements, sleep-related and morning-state indicators, and work engagement. The measures were not all developed *de novo*. Demographic items and the UWES-9 were established measures, whereas the early-morning schedule indicators and brief state-oriented sleep and fatigue items were tailored to the focal early-start episode. This contextualization was necessary because existing generic workload or sleep instruments do not jointly capture early-duty frequency, institutionally required advance arrival, teachers’ enacted advance-arrival behavior, and the prior-night and next-morning states tied to a specific early teaching episode. The following subsections therefore distinguish established instruments from context-specific indicators and describe the scoring and empirical checks used for each.

### Demographic variables

Participants reported gender, age, teaching experience, weekly teaching load, professional rank, highest degree, and administrative responsibilities (yes/no). Weekly teaching load was assessed as the self-reported number of teaching periods per week. Professional rank was reported in four ordered categories (junior, intermediate, associate senior, and full senior), and administrative responsibilities were reported as a binary role indicator. These variables were used to describe the sample and, where relevant, to test role-related heterogeneity or to serve as covariates in robustness analyses. The corresponding questionnaire items included age, years of teaching experience, weekly teaching load, rank, degree, and whether the respondent held administrative responsibilities.

### Early-morning teaching burden and time arrangements

To capture early-morning burden beyond simple early-class counts, we constructed an early-morning time pressure index integrating three indicators: (a) early-morning duty frequency in the past week (workload), (b) institutional advance-arrival requirements on 8 a.m. teaching days, measured in minutes (sysadv), and (c) teachers’ actual advance-arrival time on 8 a.m. teaching days, also measured in minutes (actuadv). Participants reported workload as the number of days in the past week on which they had undertaken an early-morning duty. For sysadv and actuadv, respondents reported how many minutes before the start of the early class they were required to arrive and how many minutes before the class they typically arrived, respectively. In the questionnaire, these two items were explicitly asked in minutes.

The index was specified as a composite (formative) indicator because these components jointly constitute an early-morning time ecology that combines structural constraints with enacted preparation costs. Because the three indicators are expressed in different units, each was standardized prior to aggregation (OECD, 2008). Specifically, z-scores were created for workload, sysadv, and actuadv, and the index was computed as their mean, Index = mean (Zworkload, Zsysadv, and Zactuadv) (Greco et al., 2019), with higher values indicating greater time pressure.

We conceptualize actuadv as an enacted temporal cost: time allocated to earlier campus presence reduces the limited window available for sleep and other recovery activities, regardless of its motivational source (Hobfoll, 1989). Empirical justification for aggregation was evaluated using bivariate correlations and principal component analysis, consistent with relying on convergence evidence rather than internal consistency estimates for reflective multi-item scales (Hair et al., 2019).

### Sleep-related indicators

Subjective sleep quality was assessed with a single state-oriented item asking teachers to rate their sleep quality for the night preceding the focal early-morning teaching episode, on a 5-point scale with higher scores indicating better perceived sleep quality (Dietch et al., 2019). Because respondents were instructed to recall the most typical early-morning teaching situation during the previous week, the sleep-quality item refers to the prior night associated with that focal episode rather than to sleep in general. In the questionnaire, this item was phrased as “How was your sleep quality last night?” and appeared within the focal-episode section.

This item was designed to capture a proximal, day-relevant recovery appraisal aligned with early-morning schedule pressures and next-morning functioning (Dietch et al., 2019). Because sleep quality was measured with a single state-oriented item, we report its scoring direction and analytic role in the model rather than evaluating it as a multi-item scale requiring internal consistency estimates (Fisher et al., 2016).

Sleep duration (sleeptim) was derived from respondents’ reported bedtime on the previous night and wake-up time on the focal morning. The resulting value was converted into hours and used as a robustness covariate rather than as a focal construct. Thus, in the present study, sleep quality indexes a proximal recovery appraisal, whereas sleeptim reflects sleep quantity associated with the same early-morning episode. The questionnaire collected these data through reported clock times for going to sleep and waking up.

### Morning fatigue

Morning fatigue was operationalized as a brief two-item composite designed to capture momentary depletion associated with the focal early-morning teaching episode. Respondents assessed (a) their level of morning sleepiness and (b) whether they felt lacking in energy during class, with both items referring to the same recalled early-start day. In the questionnaire, morning sleepiness was assessed by the item “How sleepy did you feel this morning?” and low energy was captured by the item “Did you feel lacking in energy during class today?”

To ensure directional consistency, the energy item was scored such that higher values reflected greater energy insufficiency (i.e., lower vitality). Consequently, higher scores on the resulting index indicate greater fatigue. The two indicators showed a meaningful positive correlation (r = 0.472, *p* < 0.001, N = 516), corresponding to a Spearman–Brown reliability estimate of 0.64, which supports their aggregation into a brief but coherent morning depletion composite (Eisinga et al., 2013). This parsimonious approach was intended to capture both drowsiness and low energy, conceptually distinct from the prior-night appraisal of sleep quality (Shen et al., 2006). Thus, higher fatigue scores consistently indicate greater morning depletion during the focal early-morning work episode.

### Work engagement

Work engagement was assessed using the Chinese-language UWES-9, which was included in the focal-episode section of the questionnaire (Schaufeli et al., 2006). The Chinese item wording was based on the Chinese adaptation of the UWES developed and validated by Zhang and Gan (2005). Their validation study among Chinese middle-school teachers supported the three-factor structure of work engagement—vigor, dedication, and absorption—and demonstrated acceptable internal consistency and construct validity. In the present study, the nine items were rated on a 7-point scale and summed to yield a total score ranging from 9 to 63, with higher scores indicating greater work engagement. A sample item is “I am enthusiastic about my job.” The total UWES-9 score served as the primary occupational outcome in the mediation and moderation analyses and showed good internal consistency in the present sample (Cronbach’s *α* = 0.85).

### Statistical analysis

All analyses were conducted using SPSS 22.0 with the PROCESS macro (Version 4.2) (Hayes, 2022). We first computed descriptive statistics for the focal variables and examined Pearson correlations among workload, sysadv, and actuadv. A principal component analysis was then conducted to assess empirical convergence among these three indicators and to justify their aggregation into a composite early-morning time pressure index.

Next, we estimated a serial mediation model using PROCESS Model 6, with Index as the predictor (X), subjective sleep quality (sleepqua) as the first mediator (M1), morning fatigue (fatigue) as the second mediator (M2), and work engagement (UWES) as the outcome (Y). Indirect effects were evaluated using percentile bootstrap confidence intervals based on 5,000 resamples (95% CI) (Preacher and Hayes, 2008). Because the design was cross-sectional and based on self-report data, all mediation findings were interpreted as statistical associations rather than causal effects (Maxwell and Cole, 2007).

To examine boundary conditions, we conducted moderation and moderated mediation analyses. PROCESS Model 7 was used to test whether the association between Index and fatigue varied across age, teaching experience, professional rank, and administrative responsibilities, with each moderator tested in a separate model. PROCESS Model 14 was used to test whether administrative responsibilities further moderated the association between fatigue and work engagement. Administrative responsibilities were coded 1 = yes, 2 = no. Age and teaching experience were treated as continuous variables, whereas professional rank was treated as an ordered categorical indicator (1–4), such that rank-related moderation was interpreted as a linear-by-rank trend rather than as nominal group contrasts.

In line with recommendations to avoid unnecessary overcontrol in process models, the primary analyses were estimated without covariates so that the focal associations could be interpreted parsimoniously. Demographic variables were then incorporated in robustness checks to assess whether the main findings were sensitive to sample composition. Specifically, covariate-adjusted models controlled for gender, age, degree, professional rank, and teaching experience, and an additional robustness specification further controlled for sleep duration (sleeptim). These robustness results are reported in the Appendix. We did not include additional unmeasured background factors, such as marital status, because such variables were not available in the dataset; this is acknowledged as a limitation.

Because the focal index was specified as a composite indicator and the sleep/fatigue measures were intentionally brief state indicators, CFA was not considered the most appropriate validation strategy for these measures.

## Results

### Participants and early-morning teaching context

A total of 516 valid responses from in-service university faculty were included in the analyses. The demographic characteristics of the sample are summarized in Table 1. The sample was relatively balanced by gender (male: 239, 46.3%; female: 277, 53.7%). Participants were, on average, 36.19 years old (SD = 5.53) and reported 8.23 years of teaching experience on average (SD = 5.06). Their weekly teaching load averaged 9.00 teaching periods per week (SD = 3.70). In terms of educational background, 305 teachers held a bachelor’s degree (59.1%), 122 a master’s degree (23.6%), and 89 a doctoral degree (17.2%). Professional ranks were distributed as follows: teaching assistant (153, 29.7%), lecturer (249, 48.3%), associate professor (98, 19.0%), and professor (16, 3.1%). A total of 119 teachers (23.1%) reported holding administrative responsibilities, whereas 397 (76.9%) did not. The frequency of early-morning teaching duties is also shown in Table 1.

**Table 1:** Demographic characteristics of the participants (*N* = 516).

Variables	Categories	N (%)
Gender	Male	239 (46.3)
	Female	277 (53.7)
Degree	Bachelor	305 (59.1)
	Master	122 (23.6)
	Doctoral	89 (17.2)
Rank	Teaching assistant	153 (29.7)
	Lecturer	249 (48.3)
	Associate Professor	98 (19.0)
	Professor	16 (3.1)
Admin role	Yes	119 (23.1)
	No	397 (76.9)
Frequency (early duty)	2 days	48 (9.3)
	3 days	197 (38.2)
	4 days	149 (28.9)
	5 days	55 (10.7)
	6–7 days	67 (13.0)
	
	

Age: M = 36.19, SD = 5.53, Teaching experience: M = 8.23, SD = 5.06, Weekly teaching load: M = 9.00, SD = 3.70.

Early-morning teaching duties were common in this sample. Teachers reported between 2 and 7 early-morning duty days in the previous week. The distribution was 2 days (48, 9.3%), 3 days (197, 38.2%), 4 days (149, 28.9%), 5 days (55, 10.7%), and 6–7 days (67, 13.0%), indicating substantial variability in early-morning exposure within a faculty population for whom 8 a.m. teaching appeared to be a routine part of weekly work schedules. Mean age, teaching experience, and weekly teaching load are also reported in Table 1 for descriptive completeness.

### Descriptive statistics

Descriptive statistics for the focal variables are presented in Table 2. Teachers reported an average of 3.81 early-morning duty days per week (SD = 1.18). Institutional advance-arrival requirements (sysadv) averaged 11.98 min (SD = 5.63), and teachers reported arriving 15.17 min early on average (actuadv; SD = 6.35). Sleep duration (sleeptim) averaged 7.48 h (SD = 0.79), subjective sleep quality (sleepqua) averaged 3.65 (SD = 1.07), morning fatigue (fatigue) averaged 3.32 (SD = 1.33), and work engagement (UWES) averaged 48.97 (SD = 7.07). Together, these descriptives indicate meaningful between-person variation in both early-morning scheduling demands and proximal functioning states.

**Table 2:** Descriptive statistics and correlations among study variables.

Variables	Mean	SD	1	2	3	4	5	6	7
1. Workload (Frequency)	3.810	1.180	-						
2. SysAdv (Requirement)	11.979	5.630	0.305**	-					
3. ActuAdv (Behavior)	15.167	6.350	0.253**	0.470**	-	-			
4. Early-morning time pressure index	0.000	0.750	0.693**	0.789**	0.766**				
5. Sleep quality	3.647	1.072	0.177**	0.076	0.061	0.139**	-		
6. Morning fatigue	3.320	1.327	−0.014	−0.099*	−0.185**	−0.133**	−0.247**	-	
7. Work engagement	48.97	7.07	0.118**	−0.230**	−0.104*	−0.096*	0.329**	−0.329**	-

*N* = 516. *: *p* < 0.05, **: *p* < 0.01. Workload, SysAdv, and ActuAdv are the three components of the early-morning time pressure index. Morning fatigue is a two-item composite; the two items correlated at r = 0.47 (*p* < 0.001). Internal consistency coefficients are reported only for multi-item scales (e.g., UWES).

As shown in Table 2, the three observed components of early-morning time pressure were significantly intercorrelated: workload correlated with sysadv (r = 0.31, *p* < 0.01) and actuadv (r = 0.25, *p* < 0.01), and sysadv correlated with actuadv (r = 0.47, *p* < 0.01). Sleep quality was positively associated with work engagement (r = 0.33, *p* < 0.01) and negatively associated with fatigue (r = −0.25, *p* < 0.01). Morning fatigue showed the strongest bivariate association with work engagement (r = −0.45, *p* < 0.01). These correlations are consistent with the proposed process model while also indicating that the three index components retain distinct but related informational value.

### Construction and preliminary validation of the early-morning time pressure index

To represent early-morning time pressure more comprehensively, we integrated three indicators capturing workload exposure and time-advance demands: early-morning duty frequency in the past week (workload), institutional advance-arrival requirements (sysadv), and teachers’ actual advance-arrival time (actuadv). The significant intercorrelations reported in Table 2 suggested convergence among the structural and behavioral components of early-morning burden. A principal component analysis provided further support for aggregation. As shown in Table 3, a single component was extracted, explaining 56.49% of the variance (eigenvalue = 1.695). Component loadings were 0.644 for workload, 0.814 for sysadv, and 0.785 for actuadv, with communalities of 0.415, 0.663, and 0.617, respectively. These results indicate that the three indicators captured a common underlying early-morning time pressure dimension, supporting the use of a composite index in subsequent mediation and moderation analyses.

**Table 3:** Factor loadings for the early-morning time pressure index.

Indicators	Factor loadings	Communalities
Workload	0.644	0.415
SysAdv	0.814	0.663
ActuAdv	0.785	0.617
Eigenvalue	1.695	
Variance explained	56.49%	

The resulting index showed adequate dispersion in this sample (M = 0.00, SD = 0.75, range −1.46 to 2.99), consistent with meaningful individual differences in experienced early-morning time pressure. Because the index was conceptualized as a composite rather than a reflective scale, this convergence evidence was treated as pragmatic support for aggregation rather than as evidence of latent-scale unidimensionality.

### Serial mediation linking early-morning time pressure to work engagement

**Figure 2:**
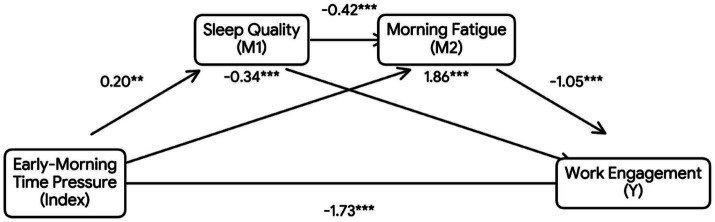
Serial mediation model with unstandardized coefficients.

We tested a serial mediation model (PROCESS Model 6) to examine whether early-morning time pressure (Index) was associated with teachers’ work engagement (UWES) through subjective sleep quality (sleepqua) and morning fatigue (fatigue). The specific path coefficients are illustrated in Figure 2, whereas the direct and indirect effect estimates are summarized in Table 4. Full model estimates across specifications are reported in Supplementary Table S1.

**Table 4:** Serial mediation analysis of early-morning time pressure on work engagement.

Pathways	Effect (B)	Boot SE	95% CI lower	95% CI upper
Total Indirect Effect	0.818	0.189	0.487	1.230
Ind1: Index → sleep quality → engagement	0.369	0.129	0.151	0.656
Ind2: Index → fatigue → engagement	0.360	0.109	0.176	0.603
Ind3: Index→sleep quality→fatigue→engagement	0.089	0.035	0.034	0.167
Direct effect				
Index → engagement	−1.726	0.386	−2.484	−0.968

Higher early-morning time pressure was significantly associated with better-rated sleep quality (B = 0.199, SE = 0.063, t = 3.184, *p* = 0.002). In the model predicting fatigue, early-morning time pressure was also a significant correlate (B = −0.342, SE = 0.092, t = −3.734, *p* < 0.001), and sleep quality was significantly associated with lower fatigue (B = −0.425, SE = 0.064, t = −6.632, *p* < 0.001). In the final equation predicting work engagement, the direct association between Index and UWES remained significant (B = −1.726, SE = 0.386, t = −4.473, *p* < 0.001). Both mediators were also significantly related to engagement: sleep quality was positively associated with UWES (B = 1.856, SE = 0.277, t = 6.691, *p* < 0.001), whereas fatigue was negatively associated with UWES (B = −1.054, SE = 0.184, t = −5.739, *p* < 0.001).

Bootstrapping (5,000 samples) indicated a significant total indirect effect of Index on UWES (Effect = 0.818, BootSE = 0.189, 95% CI [0.487, 1.230]). The specific indirect effects were also significant for the pathway through sleep quality alone (Effect = 0.369, 95% CI [0.151, 0.656]), through fatigue alone (Effect = 0.360, 95% CI [0.176, 0.603]), and through the serial pathway Index → sleep quality → fatigue → UWES (Effect = 0.089, 95% CI [0.034, 0.167]). Together, these results indicate that sleep-related appraisal and morning fatigue were statistically implicated in the association between early-morning time pressure and work engagement.

To aid interpretation, higher sleepqua scores indicate better perceived sleep quality, whereas higher fatigue scores indicate greater morning tiredness and energy insufficiency. The coexistence of positive indirect effects and a negative direct association suggests an inconsistent or competitive mediation pattern at the association level. This configuration is consistent with the composite specification of the Index, which integrates both structural time constraints (sysadv) and enacted preparatory investment (actuadv). Accordingly, higher Index values may reflect both institutional time costs and compensatory preparation efforts, yielding offsetting association patterns in cross-sectional data. Covariate-adjusted robustness checks, including additional control for sleep duration, are reported in the Appendix; the substantive pattern of the sleep-related and serial indirect effects remained unchanged.

### Boundary-condition analyses

To clarify heterogeneity in early-morning schedule sensitivity, we conducted moderation and moderated mediation analyses. In the primary models, age, teaching experience, professional rank, and administrative responsibilities were each tested separately as moderators of the Index → fatigue path using PROCESS Model 7. Administrative responsibilities were also examined as a moderator of the fatigue → UWES path using PROCESS Model 14. Detailed covariate-adjusted robustness checks are presented in Supplementary Tables S2–S4.

### Age

Age showed weaker and less robust moderation evidence than professional rank and administrative responsibilities. In the adjusted model, the Index × age interaction was attenuated and no longer reached conventional significance (B = −0.034, SE = 0.019, *p* = 0.0769, 95% CI [−0.071, 0.004]), although the conditional effects remained directionally consistent with a stronger Index → fatigue association at higher age levels. The corresponding index of moderated mediation in the adjusted model was small and not statistically significant (Index = 0.045, BootSE = 0.025, 95% CI [−0.000, 0.099]; see Table S4). A simple-slopes visualization of this pattern is provided in Supplementary Figure S1. As summarized in Supplementary Table S4, this pattern remained at trend level after additional control for sleep duration.

#### Teaching experience

Teaching experience did not show a moderating role. The Index × teaching experience interaction predicting fatigue was not significant in either the unadjusted or adjusted models (see Supplementary Table S4), and the corresponding index of moderated mediation was also not significant. This null pattern remained unchanged after additional control for sleep duration, suggesting that heterogeneity in schedule sensitivity was more strongly tied to role structure and career positioning than to years of service alone.

#### Professional rank

Professional rank showed robust boundary-condition evidence. Because rank was treated as an ordered categorical indicator of seniority, the interaction is interpreted as a linear-by-rank trend across ordered career positions. The Index × rank interaction predicting fatigue was significant in both the unadjusted and adjusted models (see Supplementary Table S4), and conditional effects indicated that the Index → fatigue association was weak or absent at lower ranks but became progressively stronger at higher ranks. The corresponding index of moderated mediation was significant, supporting rank-contingent differences in the fatigue-related indirect pathway. A simple-slopes visualization of this moderation pattern is presented in Supplementary Figure S2.

#### Administrative responsibilities

Administrative responsibilities exhibited the most consistent and practically consequential boundary evidence. In PROCESS Model 7, the Index × manage interaction predicting fatigue was significant (B = −0.750, SE = 0.208, t = −3.597, *p* < 0.001), accounting for an incremental R^2^ of 0.0232 (*F* = 12.936, p < 0.001). Conditional effects indicated that the Index → fatigue association was not significant among teachers with administrative roles (manage = 1; B = 0.177, *p* = 0.309) but was significant among those without such roles (manage = 2; B = −0.573, *p* < 0.001). The corresponding index of moderated mediation was significant (Index = 1.049, BootSE = 0.268, 95% CI [0.574, 1.621]). These detailed results are summarized in Supplementary Table S2.

**Figure 3:**
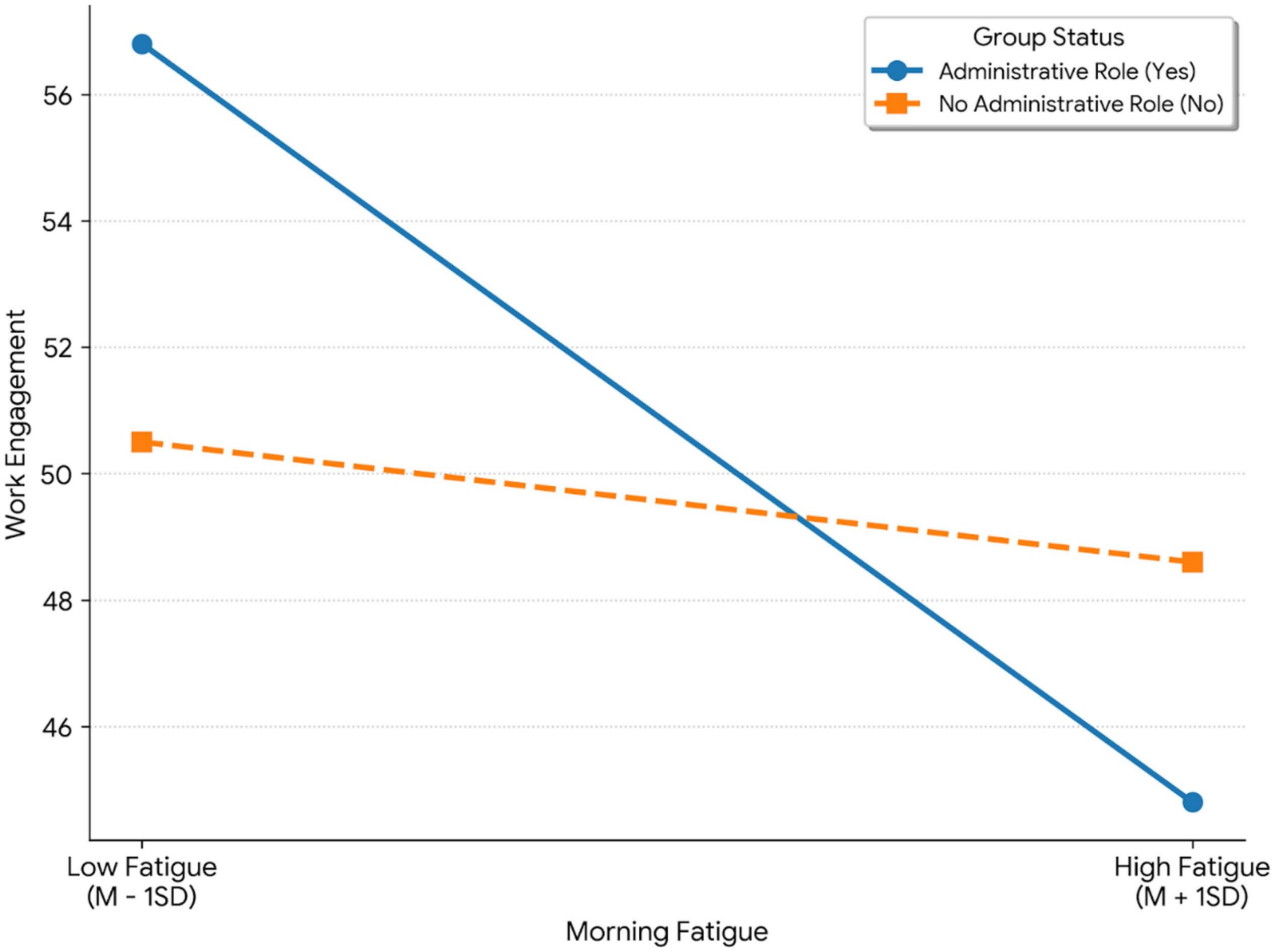
The moderating role of administrative responsibilities.

In PROCESS Model 14, administrative responsibilities also moderated the fatigue → UWES association. The fatigue × manage interaction was significant (B = 4.057, SE = 0.463, t = 8.7618, *p* < 0.001), with an R^2^ change of 0.1157 (*F* = 76.769, p < 0.001). Fatigue was much more strongly and negatively associated with engagement among teachers with administrative roles (manage = 1; B = −4.789, *p* < 0.001) than among teachers without such roles (manage = 2; B = −0.732, *p* < 0.001). Consistently, the conditional indirect effect of early-morning time pressure on UWES via fatigue was significant in both groups but substantially larger for administrative faculty (manage = 1: Effect = 2.041, 95% CI [1.243, 2.988]; manage = 2: Effect = 0.312, 95% CI [0.119, 0.552]). The index of moderated mediation was also significant (Index = −1.729, BootSE = 0.405, 95% CI [−2.598, −1.031]). These results are visualized in Figure 3 and summarized in Table S3.

Taken together, the moderation analyses indicate that the consequences of early-morning time pressure are not uniform across teachers. Rather, role structure and career positioning appear to shape both the strain-related implications of schedule demands and the extent to which fatigue translates into lower engagement.

#### Robustness checks and alternative specifications

To assess robustness, we re-estimated the focal models under three specifications: (a) the parsimonious models reported in the main text, (b) models adjusted for demographics (gender, age, degree, rank, and teaching experience), and (c) models further adjusted for sleep duration (sleeptim). Across specifications, the core serial mediation pattern remained stable. As shown in Supplementary Table S1, Index predicted sleep quality in all models (B ≈ 0.18–0.20, *p* < 0.01), sleep quality predicted lower fatigue (B ≈ −0.38 to −0.42, *p* < 0.001), and fatigue predicted lower engagement (B ≈ −1.01 to −1.05, *p* < 0.001). The total indirect effect remained significant across models (≈ 0.75–0.82), and the serial indirect pathway (Index → sleep quality → fatigue → engagement) remained significant in all three specifications.

For the moderation analyses, the Index × manage interaction predicting fatigue remained significant across all specifications (Bint ≈ − 0.73 to −0.75), with consistently negative conditional effects for non-administrative teachers and non-significant effects for administrative teachers (see Supplementary Table S2). Likewise, the fatigue × manage interaction predicting engagement remained robust (Bint ≈ 4.01–4.06), and the fatigue–engagement slope remained substantially more negative among administrative teachers than among non-administrative teachers (see Supplementary Table S3). Overall, these checks indicate that the central sleep-related/serial pathway and the role-contingent fatigue cost pattern were not driven by demographic composition or sleep duration differences.

## Discussion

### Overview of findings

Building on the schedule-alignment perspective developed in student research, the present study shifts attention to the teacher side of early schedules (Yeo K. et al., 2023; Yeo et al., 2023a; Yeo et al., 2023b). In Chinese universities, 8 a.m. classes are embedded in routines that often require advance arrival and intensified pre-class preparation. We therefore conceptualized early teaching burden as early-morning time pressure and operationalized it using a composite index integrating (a) early-duty frequency, (b) institutional advance-arrival requirements, and (c) teachers’ actual advance-arrival behavior. Because the index combines structural constraints with enacted preparatory investment, it may reflect a joint ecology of institutional time cost and proactive morning coping rather than a purely “cost-only” burden.

Empirically, the three indicators converged, with significant intercorrelations and a dominant first component, supporting an index-based representation beyond simple early-class counts. In JD-R terms, the index represents a temporal job demand, sleep quality and morning fatigue represent proximal recovery-related resource states, and work engagement represents a motivational outcome. Teachers facing greater early-morning time pressure tended to report lower work engagement, but the overall pattern was not explained by a single pathway. Rather, prior-night sleep appraisal and morning fatigue were statistically implicated in offsetting indirect pathways, while a negative direct association remained. Boundary-condition analyses further revealed meaningful heterogeneity: the time pressure–fatigue link showed clearer evidence of variation by professional rank, and weaker but directionally similar variation by age. Collectively, these results support a teacher-centered, role-sensitive extension of both schedule-alignment research and JD-R theorizing in higher education.

### Interpreting the sleep–fatigue pathway

The joint involvement of sleep quality and fatigue provides a coherent bridge between early institutional time structures and teachers’ engagement. Within the JD-R framework, early-morning time pressure can be viewed as a temporal demand that draws on recovery opportunities before the workday begins, whereas perceived sleep quality and morning fatigue reflect proximal resource preservation or depletion. At the association level, better perceived sleep quality aligned with lower morning fatigue, which in turn aligned with higher engagement. The negative fatigue–engagement association is therefore consistent with a resource-depletion process adjacent to the JD-R health-impairment pathway: when energetic resources are reduced, sustaining vigor, dedication, and absorption becomes more difficult (Bakker and de Vries, 2021; Kühnel et al., 2017).

However, the positive association between the early-morning index and sleep quality warrants cautious interpretation. Because the index integrates both structural constraints, such as advance-arrival mandates, and enacted preparation investment, namely actual early arrival, higher index values may partly capture teachers’ anticipatory adjustment to predictable early demands. For example, teachers may make anticipatory adjustments to accommodate early demands, such as earlier bedtimes, to ensure punctuality and next-morning readiness, which could be reflected in higher-rated sleep quality and lower fatigue. Importantly, such adjustment does not imply that time pressure is beneficial; rather, it may indicate short-term compensation that preserves basic recovery appraisals under constrained schedules.

Consistent with this view, the coexistence of positive indirect pathways via sleep-related indicators and a remaining negative direct association with engagement is consistent with a possible compensatory pattern at the association level: sleep-related appraisals and fatigue capture one part of the early-morning experience, whereas other unmeasured processes—such as reduced time autonomy, constrained evening recovery experiences, or morning affective states—may continue to link time pressure to lower occupational vitality (Arnold and Sonnentag, 2023; Hockey, 1997; Sonnentag et al., 2020). Because we did not directly measure coping strategies, recovery experiences, or pre-sleep affect, these interpretations should be treated as plausible explanations rather than definitive mechanisms.

### Boundary conditions and subgroup patterns

The moderation findings suggest that early-morning time pressure is not an equal-opportunity stressor. Its association with fatigue appears sensitive to role structure and career positioning. Age moderated the time pressure–fatigue link, with conditional estimates suggesting a stronger association at higher age levels. This does not imply lower coping capacity among older teachers; rather, early scheduling may carry different implications across life and career stages due to routines, responsibilities, and recovery contexts (Kooij et al., 2020).

Professional rank also moderated the time pressure–fatigue association, with stronger links at higher rank levels. One plausible interpretation is that seniority entails greater role salience and layered responsibilities, making early-morning time structures more consequential for daily energy states (Mudrak et al., 2018).

Administrative responsibilities provided the most practically consequential boundary evidence. Administrative status conditioned the time pressure–fatigue association and also moderated the fatigue–engagement link: fatigue was much more strongly associated with lower engagement among teachers with administrative roles. From a JD-R perspective, this pattern reflects demand accumulation: early teaching is layered with coordination, accountability, and decision-making requirements, while the available recovery and scheduling resources may not increase correspondingly. When instructional and managerial demands are coupled in the same morning window, fatigue may therefore carry greater motivational and attentional costs for sustaining engagement (Floyd, 2016). By contrast, teaching experience did not show a clear moderating role, indicating that heterogeneity may be more closely tied to role structure than to tenure alone (Toropova et al., 2021). Put differently, morning fatigue appears to be especially costly for teachers who must simultaneously manage instructional and administrative responsibilities.

### Theoretical implications

This study contributes to the evolving literature on institutional schedules, sleep-related functioning, and educational sustainability in several ways.

First, it extends early-start research beyond students to address the teacher side of schedule alignment, specifically by contextualizing the Job Demands-Resources (JD-R) framework within the temporal ecology of higher education (Demerouti et al., 2001). While Yeo’s findings highlight how early classes can shape students’ sleep opportunities and academic engagement (Yeo et al., 2023a), our results suggest that early schedules function as distinct job demands for faculty that are meaningfully linked to occupational vitality. Specifically, sleep quality and fatigue represent plausible proximal indicators of a resource-depletion process initiated by these temporal pressures. This extension matters because the sustainability of early schedules depends not only on student outcomes but also on the functional readiness of those delivering instruction.

Second, by constructing and validating an early-morning time pressure index, the present study offers a measurement advance. Early teaching has often been operationalized as a dichotomous marker or a simple frequency count (Skaalvik and Skaalvik, 2011). The convergence of frequency, institutional requirements, and actual advance-arrival behavior indicates that early-morning burden can be represented as a more integrated dimension that better reflects real-world governance and preparation demands.

Third, the pattern of significant indirect effects alongside a remaining direct association points to a layered mechanism landscape. In JD-R terms, sleep quality and fatigue appear to capture meaningful recovery-related resource states, but they do not exhaust the demands and resources through which early schedules may shape engagement. Future expansions could incorporate perceived time autonomy, schedule flexibility, organizational support, morning affect, and pre-class efficacy to clarify which additional job resources buffer—and which additional demands intensify—the residual association between time pressure and engagement (Albrecht, 2010).

Finally, moderation evidence suggests that teacher-centered schedule-alignment frameworks in higher education should be explicitly role-contingent. Rather than assuming uniform sensitivity to early schedules, future models should incorporate heterogeneity linked to career positioning and institutional responsibilities (Bentley et al., 2013).

From a work and organizational psychology perspective, the present findings contribute to a growing literature treating work timing and temporal constraints as job demands that compress recovery opportunities and shape day-level functioning. Consistent with effort–recovery and JD–R theorizing, early starts can reallocate time from nonwork recovery to prework effort, increasing vulnerability to morning depletion and downstream motivational states such as engagement (Demerouti et al., 2001; Sonnentag and Fritz, 2007). Our index-based operationalization extends this perspective by integrating both structural constraints, namely institutional advance-arrival requirements, and enacted time investment, namely actual advance arrival, thereby capturing a time–pressure ecology rather than a single “early class count.” In addition, emerging work highlights that engagement and well-being show temporal patterning and can vary meaningfully across daily and weekly cycles (Wang, 2024), suggesting that institutional scheduling practices represent a consequential, yet under-theorized, lever for work design in academic settings.

### Practical implications and institutional recommendations

Although the present findings are associative and based on self-report data, they suggest several implications for work design and scheduling practice in settings where 8 a.m. teaching is routine and accompanied by formal advance-arrival rules.

#### Decouple early teaching from administrative demand intensity

The strongest subgroup evidence indicates that administrative responsibilities amplify the engagement costs of morning fatigue. Where flexibility exists, universities should avoid scheduling demanding managerial tasks or mandatory administrative meetings on the same mornings when administrative faculty teach at 8 a.m., and consider prioritizing later teaching slots for staff with heavy administrative roles (Kinman and Johnson, 2019).

#### Shift from early-class counts to time–cost accounting

Evaluations of early schedules should consider not only how many early classes are assigned, but also mandated advance-arrival rules and teachers’ enacted preparation time, which together capture hidden time inequities that slot-count approaches may miss (Vostal, 2015).

#### Avoid consecutive early-duty stacking for the same individuals

Distributing early-morning duties more evenly across staff and across weeks may reduce concentrated time pressure within specific subgroups (Lee et al., 2025; Misra et al., 2021; Sonnentag, 2018).

#### Implement recovery-supportive work-design micro-policies

Low-cost practices, such as limiting additional early-morning non-teaching meetings on 8 a.m. teaching days and clarifying evening workload boundaries during heavy early-teaching weeks, may help protect recovery windows and reduce morning depletion risk (Bennett et al., 2018).

#### Use data-informed scheduling reviews

Periodic reviews of early-morning allocation patterns can help ensure that early schedules do not systematically accumulate among subgroups for whom fatigue-related engagement costs may be stronger (Malcom et al., 2025; O’Meara et al., 2019).

### Limitations and future directions

Several limitations should be acknowledged. First, because key variables were assessed via self-report at a single wave, the serial ordering implied by the model should be interpreted as associational rather than causal; cross-sectional mediation can be biased and cannot rule out alternative temporal sequences (Maxwell and Cole, 2007). Diary or experience-sampling designs would therefore be valuable for testing within-person dynamics and day-to-day coupling between early starts, recovery appraisals, morning fatigue, and engagement. Second, the study relied on a convenience sample, which may limit representativeness and introduce selection bias. The findings should therefore be interpreted as evidence from a multi-regional convenience sample of Chinese university teachers rather than as nationally representative estimates. Third, sleep quality was assessed with a single prior-night appraisal and fatigue with a brief two-item composite. Although these measures were intentionally state-oriented to match the day-relevant nature of early timetabling, future research should replicate the model using validated multi-item sleep and fatigue instruments and, where possible, objective indicators. Fourth, we inferred anticipatory adjustment as a plausible account of the positive association between time pressure and sleep appraisal, but coping processes were not measured directly; future work should include measures of pre-sleep affect, bedtime regulation, and recovery experiences. Fifth, we did not measure overall working hours, so the observed associations may partly reflect broader workload intensity rather than early-morning scheduling alone; future studies should jointly model schedule timing and total working hours. Future research should also incorporate family-context variables, including marital status, parenthood, and caregiving responsibilities, together with work-organization factors such as schedule autonomy, flexibility of hours, total workload, and organizational support. These variables may operate as additional demands or resources within the JD-R framework and may explain why faculty experience the same early start differently. In addition, although work engagement and morning fatigue are conceptually distinct, both include energy-related content, which may have modestly inflated their association. Finally, we did not assess chronotype or morningness–eveningness, which may shape the degree of mismatch between work timing and individual biological rhythms. Future research should therefore examine person–schedule fit more directly and test schedule–role coupling across broader organizational contexts.

## Conclusion

In a multi-regional convenience sample of Chinese university faculty, early-morning time pressure—operationalized as a composite of early-duty frequency, institutional advance-arrival requirements, and teachers’ actual advance-arrival behavior—was significantly associated with work engagement. Subjective sleep quality and morning fatigue statistically accounted for meaningful portions of this association, including a serial pathway from time pressure to sleep quality to fatigue and then to engagement. Boundary-condition tests indicated that age and professional rank shaped the time pressure–fatigue link, and administrative responsibilities emerged as a key vulnerability factor that magnified the fatigue–engagement association. Together, these findings highlight early-morning scheduling as a consequential organizational context shaping recovery-related functioning and occupational vitality, with clear role-sensitive implications for work design and scheduling practice.

## Data Availability

The raw data supporting the conclusions of this article will be made available by the authors, without undue reservation.
