# Supplementary material for: Who pays more for early starts? Early-morning time pressure, sleep-related states, and role-contingent work engagement among Chinese university teachers
Source: Front Psychol. 2026 Aug 5;17:1824905. doi: 10.3389/fpsyg.2026.1824905 (PMC13487749; doi:10.3389/fpsyg.2026.1824905)
Supplement: Supplementary file 1 [file Supplementary_file_1.docx]

**Appendix**

**S0. Supplementary Methodological Details**

Data Cleaning and Coding

The final sample included 516 valid responses after excluding cases with missing data or logical inconsistencies.

- **Administrative Responsibilities (*Manage*):** Binary (1 = Yes, 2 = No).
- **Professional Rank (*Title*):** Ordinal (1 = Teaching Assistant to 4 = Professor). Treated as a continuous predictor to test for linear trends in moderation analyses.
- **Morning Fatigue:** Composite of two items (r = .47), higher scores indicate greater fatigue.
- **Sleep Duration (*Sleeptim*):** Self-reported sleep duration in hours, used as a robustness covariate.

**Model Specification for Robustness Checks**

- **Model (a) Unadjusted:** The primary model as reported in the main text (parsimonious).
- **Model (b) Adjusted:** Controlling for gender, age, degree, professional rank, and teaching experience.
- **Model (c) Adjusted + Sleep Duration:** Additionally controlling for sleep duration (*Sleeptim*) to ensure that the effects of sleep quality and fatigue are not confounded by mere sleep quantity.

**Table S1. Serial Mediation Analysis (PROCESS Model 6) across Model Specifications**

This table demonstrates that the serial mediation pathway (Index → Sleep Quality → Fatigue → Engagement) remains robust after controlling for demographics and sleep duration.

| **Pathways** | **(a) Unadjusted**  **(Main Text)** | **(b) Adjusted**  **(w/ Demographics)** | **(c) Adjusted**  **(w/ Demo + Sleeptim)** |
| --- | --- | --- | --- |
| **Direct Paths (Unstandardized B)** |  |  |  |
| Index → Sleep Quality (a_1_) | 0.20** | 0.20** | 0.18** |
| Index → Fatigue (a_2_) | -0.34*** | -0.31** | -0.30** |
| Sleep Quality → Fatigue (d_21_) | -0.42*** | -0.42*** | -0.38*** |
| Fatigue → Engagement (b_2_) | -1.05*** | -1.03*** | -1.01*** |
| Index → Engagement (c') | -1.73*** | -1.58*** | -1.55*** |
| **Indirect Effects (Bootstrap 95% CI)** | **Effect [LLCI, ULCI]** | **Effect [LLCI, ULCI]** | **Effect [LLCI, ULCI]** |
| **Total Indirect Effect** | **0.82 [0.49, 1.23]** | **0.77 [0.45, 1.17]** | **0.75 [0.42, 1.15]** |
| Ind1: Index → Sleep → UWES | 0.37 [0.15, 0.66] | 0.36 [0.14, 0.65] | 0.34 [0.12, 0.61] |
| Ind2: Index → Fatigue → UWES | 0.36 [0.18, 0.60] | 0.32 [0.15, 0.55] | 0.30 [0.13, 0.52] |
| **Ind3: Serial (Index** → **Sleep** → **Fatigue** → **UWES)** | **0.09 [0.03, 0.17]** | **0.09 [0.03, 0.17]** | **0.07 [0.02, 0.14]** |

*Note.* N=516. **Model (b)** covariates: Gender, Age, Degree, Rank, Teaching Experience. **Model (c)** covariates: Model (b) + Sleep Duration. *p<.05, **p<.01, ***p<.001.

**Table S2. Moderation of the Index** → **Fatigue Link by Administrative Responsibilities (Model 7)**

This table focuses on the interaction between Early-Morning Time Pressure and Administrative Responsibilities, showing that the buffering effect (or lack of association) for administrative staff is consistent across all specifications.

| **Parameter** | **(a) Unadjusted**  **(Main Text)** | **(b) Adjusted**  **(w/ Demographics)** | **(c) Adjusted**  **(w/ Demo + Sleeptim)** |
| --- | --- | --- | --- |
| **Interaction Term** |  |  |  |
| Index × Manage (B_int_) | **-0.75*** | **-0.75*** | **-0.73**** |
| SE | 0.21 | 0.21 | 0.22 |
| 95% CI | [−1.16, −0.34] | [−1.17, −0.33] | [−1.16, −0.30] |
| Δ R^2^ due to interaction | .023*** | .023*** | .020** |
| **Conditional Effects of Index on Fatigue** |  |  |  |
| Non-Administrative (Manage=2) | -0.57*** | -0.57*** | -0.55*** |
| Administrative (Manage=1) | 0.18^ns^ | 0.18^ns^ | 0.18^ns^ |

*Note. ns = not significant. The interaction suggests a differential association pattern across roles; potential explanations (e.g., anticipatory adjustment) remain speculative and should be tested in future studies.*

**Table S3. Moderated Mediation of Fatigue** → **Engagement by Administrative Responsibilities (Model 14)**

This table summarizes the moderated mediation pattern showing that the fatigue–engagement association is substantially stronger among teachers with administrative responsibilities.

| **Parameter** | **(a) Unadjusted**  **(Main Text)** | **(b) Adjusted**  **(w/ Demographics)** | **(c) Adjusted**  **(w/ Demo + Sleeptim)** |
| --- | --- | --- | --- |
| **Interaction Term** |  |  |  |
| Fatigue × Manage (B_int_) | **4.06*** | **4.06*** | **4.01*** |
| SE | 0.46 | 0.46 | 0.47 |
| 95% CI | [3.15, 4.97] | [3.15, 4.97] | [3.10, 4.93] |
| Δ R^2^ due to interaction | .116*** | .116*** | .110*** |
| **Conditional Effects of Fatigue on UWES** |  |  |  |
| Non-Administrative (Manage=2) | -0.73*** | -0.73*** | -0.70*** |
| Administrative (Manage=1) | **-4.79*** | **-4.79*** | **-4.71*** |
| **Index of Moderated Mediation** | **-1.73** | **-1.73** | **-1.70** |
| Boot 95% CI | [-2.60, -1.03] | [-2.60, -1.03] | [-2.55, -0.98] |

*Note. The robustly significant Index of Moderated Mediation confirms that the indirect pathway (Index* → *...* → *Fatigue* → *Engagement) is conditionally stronger for administrative faculty across all models.*

**Table S4. Moderation of the Index → Fatigue Link by Age, Professional Rank, and Teaching Experience (PROCESS Model 7)**

| **Moderator** | **Specification** | **Interaction term (Bint)** | **SE** | **95% CI** | **p** | **Index of moderated mediation** | **Boot 95% CI** | **Conclusion** |
| --- | --- | --- | --- | --- | --- | --- | --- | --- |
| Age | (a) Unadjusted (Main Text) | — | — | — | .021 | — | — | Significant |
|  | (b) Adjusted (w/ Demographics) | -0.0337 | 0.0190 | [-0.0711, 0.0037] | .0769 | 0.0450 | [-0.0004, 0.0994] | Trend-level; IMM not significant |
|  | (c) Adjusted (w/ Demo + Sleeptim) | — | — | — | .070 | — | — | Trend-level |
| Professional Rank | (a) Unadjusted (Main Text) | -0.5951 | 0.1313 | [-0.8530, -0.3371] | < .001 | — | — | Significant |
|  | (b) Adjusted (w/ Demographics) | -0.5151 | 0.1271 | [-0.7648, -0.2653] | < .001 | 0.8326 | [0.4810, 1.2748] | Significant; IMM significant |
|  | (c) Adjusted (w/ Demo + Sleeptim) | — | — | — | .038 | — | — | Significant |
| Teaching Experience | (a) Unadjusted (Main Text) | -0.0334 | 0.0268 | [-0.0861, 0.0193] | .2139 | — | — | Not significant |
|  | (b) Adjusted (w/ Demographics) | -0.0214 | 0.0262 | [-0.0729, 0.0301] | .4149 | 0.0292 | [-0.0377, 0.1075] | Not significant; IMM not significant |
|  | (c) Adjusted (w/ Demo + Sleeptim) | — | — | — | .390 | — | — | Not significant |
| Note. Each moderator was tested in a separate PROCESS Model 7 analysis predicting fatigue from early-morning time pressure (Index), the moderator, and their interaction. Model (b) controlled for gender, age, degree, professional rank, and teaching experience. Model (c) additionally controlled for sleep duration (sleeptim). Bint = interaction coefficient for Index × moderator. The index of moderated mediation summarizes moderator-dependent variation in the fatigue-related indirect pathway from Index to work engagement. For some unadjusted and sleeptim-adjusted exploratory robustness models, the current export files retained significance summaries but not the full moderated-mediation coefficient blocks; these cells are therefore left blank. | | | | | | | | |

**Figures S1 and S2 provide simple-slopes visualizations for the age- and rank-based moderation patterns reported in Table S4.**

**Figure S1. Moderating role of age on the Index → Fatigue link**

**Figure S2. Moderating role of professional rank on the Index → Fatigue link**
